# Preliminary Evidence for Sex Differences in CYP2C19 Metabolic Capacity During Psychotropic Drug Treatment

**DOI:** 10.3390/genes17060718

**Published:** 2026-06-21

**Authors:** Janina Eiberger, Heike Weber, Andreas Reif, Jürgen Deckert, Sebastian Walther, Martina Hahn, Maike Scherf-Clavel

**Affiliations:** 1 Department of Psychiatry, Psychosomatics and Psychotherapy, Center of Mental Health, University Hospital of Würzburg, 97080 Würzburg, Germanyweber_h2@ukw.de (H.W.); deckert_j@ukw.de (J.D.); walther_s5@ukw.de (S.W.); scherf_m@ukw.de (M.S.-C.); 2 Department of Psychiatry, Psychosomatics and Psychotherapy, University Hospital Frankfurt, 60528 Frankfurt, Germany; reif@med.uni-frankfurt.de; 3 Institute of Clinical Epidemiology and Biometry, Julius-Maximilians-Universität Würzburg, 97080 Würzburg, Germany; 4 Department of Mental Health, Varisano Hospital Frankfurt Hoechst, 65929 Frankfurt, Germany

**Keywords:** sex differences, CYP2D6, CYP2C19, metabolic capacity, psychiatry

## Abstract

**Background/Objectives**: Sex-specific differences in the pharmacokinetics of psychotropic drugs are gaining increasing clinical relevance, but only limited data are currently available on sex-specific effects within genetically defined metabolizer phenotype categories. The objective of this study was to assess genotype-dependent sex differences in the metabolic capacity of the drug-metabolizing enzymes CYP2D6 and CYP2C19. **Methods**: Statistical analyses were performed using linear mixed-effects models with subject-level random intercepts to account for repeated therapeutic drug monitoring (TDM) measurements. Venlafaxine and risperidone were used as probe drugs to find differences in the metabolic capacity of CYP2D6 and escitalopram for CYP2C19. Pharmacokinetic surrogate parameters were the metabolite-to-parent ratio (MPR) for venlafaxine and risperidone and the dose-corrected serum concentration (CD) for escitalopram. Models included sex, metabolizer phenotype, and their interaction, adjusted for age and creatinine production rate (CPR). Sex-specific differences within phenotype groups were assessed using estimated marginal means. **Results**: Among venlafaxine samples (N = 117) and risperidone samples (N = 73), no significant sex-specific differences in MPR were observed within CYP2D6 metabolizer groups. For escitalopram samples (N = 51), a significant sex difference was observed among CYP2C19 normal metabolizers (NMs), with higher CD in males compared to females. **Conclusions**: Exploratory analyses suggested a higher metabolic capacity in CYP2C19 NM females treated with escitalopram. Due to the limited sample size, however, this finding should be considered hypothesis-generating. Future studies in larger samples are needed to corroborate whether sex and other factors modulate the metabolic capacity of CYP2C19, e.g., by epigenetic mechanisms.

## 1. Introduction

Sex-specific differences are gaining increasing importance in psychiatric pharmacotherapy and are becoming a focus of translational research and clinical decision-making. Numerous studies have shown that men and women differ in the metabolism of psychotropic drugs and in their clinical treatment response, which can affect both therapeutic efficacy and the side effect profile [1,2,3,4]. These differences are based on a complex interplay of biological factors, including differences in body composition, fat and water distribution, hormonal regulation, and renal and hepatic elimination mechanisms. In general, women report adverse drug reactions more frequently than men and have a higher risk of dose-dependent side effects [5].

Therapeutic drug monitoring (TDM) is an established tool for assessing interindividual differences in pharmacokinetics. By measuring serum concentrations, TDM enables an individualized assessment of drug exposure under real-world conditions and is firmly established in clinical routine, particularly in psychiatry [6].

Several TDM-based studies have reported higher dose-adjusted serum concentrations of psychotropic medications in women compared to men, including venlafaxine, risperidone, and escitalopram [7,8,9,10,11,12]. However, in these studies, analyses were either not adjusted for genetic variation in the cytochrome P450 enzymes (CYP) CYP2D6 and CYP2C19 [9,10,11] or available genotype information was not used to investigate sex-specific effects within genetically defined metabolizer groups [12].

A key determinant of the pharmacokinetics of numerous psychotropic drugs is the activity of CYP2D6 and CYP2C19. Both enzymes show high genetic variability, reflected in different metabolizer phenotypes and activity scores, and significantly influence serum concentrations of many antidepressants and antipsychotics [13,14,15,16,17]. Accordingly, pharmacogenetic information is increasingly being considered in personalized therapy approaches. However, it is known that actual enzyme activity is also affected by concomitant medication, smoking, inflammatory conditions, or hormonal influences [9,13,14,17].

In addition, sex-specific differences in the activity of drug-metabolizing enzymes have been reported for CYP2D6, CYP2C19, CYP3A4, and CYP1A2 [1,5,18,19]. However, data are limited, and especially for CYP2D6 and CYP2C19, findings across studies are inconsistent, and robust evidence is lacking [1,5,18,20].

Furthermore, it remains unclear whether sex-related differences in CYP2D6 and CYP2C19 metabolic capacity vary across genetically defined metabolizer groups during routine psychotropic drug treatment. Addressing this issue, the present study aims to investigate whether in adult psychiatric patients during treatment with psychotropic drugs, sex differences in CYP2D6 and CYP2C19 metabolic capacity depend on genetically defined metabolizer status. We hypothesized that the metabolite-to-parent ratio (MPR) of venlafaxine and risperidone and dose-corrected serum concentration (CD) of escitalopram as surrogates for metabolic capacity (enzyme activity) differ between male and female patients within the same genotype-derived phenotype group or activity score group of the relevant metabolizing enzyme (CYP2D6 or CYP2C19).

## 2. Materials and Methods

### 2.1. Participants

#### 2.1.1. Wuerzburg Sample

Patients treated as inpatients at the Department of Psychiatry, Psychosomatics and Psychotherapy of the University Hospital Würzburg were eligible for inclusion if genotype information and therapeutic drug monitoring (TDM) data were available. Only adults aged 18 years or older were included. CYP2D6 and CYP2C19 genotyping, as well as TDM, were carried out as part of standard clinical practice. Genotyping was performed in accordance with the recommendations of the German Commission for Genetic Diagnostics and the requirements of the German Genetic Diagnostics Act, based on written informed consent obtained from all participants. The Würzburg cohort comprised 212 patients in total. Genotype and serum concentration data were collected between January 2020 and January 2022.

#### 2.1.2. Frankfurt Sample

Adult inpatients aged ≥18 years admitted to the Department of Psychiatry, Psychosomatics and Psychotherapy at the University Hospital Frankfurt with a diagnosis of a depressive episode were included if CYP2D6 and CYP2C19 genotyping had been performed within the framework of the FACT-PGx (Feasibility, acceptance and clinical utility of PGx Testing Study) study. TDM was carried out as part of routine clinical care. Only participants of the FACT-PGx study, for whom TDM data were available, were included in the analyses. The Frankfurt cohort consisted of 104 patients. Genotyping and serum concentration assessments were conducted between January 2020 and February 2022.

### 2.2. Statistical Analysis

Statistical analyses were conducted in R v4.5.0. The analyses were restricted to drugs metabolized primarily by CYP2D6 or CYP2C19 and with a sufficient number of samples (N > 50; venlafaxine: 117 samples from 107 patients; risperidone: 73 samples from 73 patients; escitalopram: 51 samples from 40 patients).

In the venlafaxine and risperidone samples, the metabolite-to-parent drug ratio (MPR), defined as the ratio of the serum concentration of the active metabolite (ODM-venlafaxine, 9OH-risperidone) to that of the parent compound, was defined as a pharmacokinetic endpoint. MPR of these drugs is an established proxy for CYP2D6-mediated metabolic activity. In the escitalopram sample, the dose-corrected serum concentration (CD; plasma concentration divided by the daily dose) was used as the outcome variable because no routinely analyzed metabolite concentration is available for calculation of MPR. CD, therefore, reflects systemic drug exposure rather than direct metabolic conversion and is influenced by both metabolic and non-metabolic processes. Accordingly, MPR of venlafaxine and risperidone was interpreted as a surrogate marker of CYP2D6-mediated metabolic capacity, whereas CD of escitalopram was interpreted as an indirect surrogate of exposure and clearance related to CYP2C19-mediated metabolism.

Linear mixed-effects models with subject-level random intercepts to account for intra-individual correlation arising from repeated TDM measurements were used for the analyses. Fixed effects included the interaction of sex-enzyme phenotype, with adjustment for age and creatinine production rate (CPR). CPR was calculated according to O’Hanlon et al. [21] as the product of the glomerular filtration rate estimated by CKD-EPI and serum creatinine. It was used as a surrogate marker for fat-free mass (FFM), indicative of physiological differences between male and female patients.

Fixed effects were evaluated using type III analysis of variance (ANOVA) with Satterthwaite’s approximation for degrees of freedom, based on the fitted linear mixed-effects models (hypothesis test of the fitted model). To improve interpretability beyond *p*-values, estimated effects together with corresponding 95% confidence intervals are reported. Also, sex differences within individual phenotype categories were estimated using model-based contrasts (estimated marginal means within each metabolizer group using Holm-adjusted tests). If a subsample was represented by only a single observation per patient in the final dataset, the random-effects structure was not identifiable. Therefore, a fixed-effects linear model was used for these analyses.

CYP2D6 and CYP2C19 genotype-derived metabolizer phenotypes were defined by the latest Clinical Pharmacogenetics Implementation Consortium (CPIC) recommendations, including normal metabolizers (NM), intermediate metabolizers (IM), poor metabolizers (PM), rapid metabolizers (RM), and ultrarapid metabolizers (UM) [16,17,22]. Regarding CYP2D6, the phenotypes were further subdivided into the corresponding activity score (AS) subgroups. Details on CYP2D6 and CYP2C19 genotyping, as well as TDM, are provided in Supplementary Methods Section S1 and Supplementary Table S1.

Outliers (>3 standard deviations from the mean) of the respective pharmacokinetic parameters (MPR, CD) were excluded prior to analysis. MPR and CD were analyzed on their original scale and were not log-transformed prior to modeling, as model diagnostics did not indicate relevant violations of distributional assumptions. Model assumptions were assessed using visual inspection of residual plots and Q–Q plots. Patients with relevant inhibitors/inducers (moderate/strong) of the respective enzymes according to the Flockhart table [22] were excluded from the analyses.

Statistical significance was assessed using a *p*-value < 0.05. Statistical tests were grouped into predefined test families (active substance). Within these test families, the *p*-values were adjusted to control for type I errors using Bonferroni correction (venlafaxine and risperidone combined N = 2, *p* = 0.025; escitalopram N = 1, *p* = 0.05). For model-based sex-specific contrasts, *p*-values were adjusted using the Holm method.

## 3. Results

### 3.1. Patient Sample

The final dataset, including patients treated with venlafaxine, risperidone, and escitalopram, comprised 234 samples, including 166 from Würzburg and 68 from Frankfurt, corresponding to a total of 179 patients.

The mean age was 46.3 ± 15.03 years (mean ± standard deviation), and 52% (N = 93) were female. 76 patients were non-smokers, 58 were smokers, and smoking status was not available for 45 patients.

Mean serum creatinine was 0.91 ± 0.2 mg/dL, and mean estimated glomerular filtration rate (CKD-EPI) was 87.6 ± 17.6 mL/min/1.73 m^2^. Patients received between 0 and 16 concomitant medications, with a mean of 5.23 ± 3.5.

Regarding CYP2D6 phenotype distribution, 53.1% (N = 95) of patients were classified as NM, 31.3% (N = 56) as IM, 4.5% (N = 8) as PM, and 2.2% (N = 4) as UM. For 16 patients (8.9%), no CYP2D6 genotype could be determined due to gene duplications with unclear allele assignment or functionally insufficiently characterized alleles. CYP2D6-relevant concomitant medication (moderate/strong inhibitors) was present in 6.8% (N = 12) of the patients.

For CYP2C19, 44.7% (N = 80) were NM, 21.8% (N = 39) IM, 0.6% (N = 1) PM, 29.1% (N = 52) RM, and 3.9% (N = 7) UM. CYP2C19-relevant concomitant medication was rare, with inhibitors or inducers identified in 2.2% (N = 4) of the patients.

A detailed overview of patient-level baseline characteristics is presented in Table 1. Demographic sample-level characteristics of the drug subgroups (venlafaxine, risperidone, escitalopram) are summarized in Table 2.

### 3.2. Venlafaxine

For patients treated with venlafaxine, a detailed overview of the MPR in male and female patients, stratified by phenotype, is presented in Table 3. Detailed results for the CYP2D6 activity score subgroup are provided in Supplementary Table S2.

In the analysis including the CYP2D6 phenotype, the random intercept variance indicated substantial between-subject variability (variance = 9.45, SD = 3.07), which exceeded the residual variance (variance = 1.02, SD = 1.01), supporting the inclusion of a mixed-effects modeling approach. Type III ANOVA revealed a significant main effect of CYP2D6 phenotype (F(3, 70.2) = 10.0, *p* < 0.001), whereas no significant main effects of sex, age, or CPR were observed. The interaction between sex and CYP2D6 status was not significant. Examination of the fixed-effect estimates indicated that, compared with NM, IM exhibited a significantly lower MPR (β = −4.36, *p* < 0.001), as well as PM (β = −4.11, *p* = 0.04). No significant differences were observed for UM. Consistent with the ANOVA results, neither sex nor sex-by-genotype interaction terms reached statistical significance. Sex-specific pairwise comparisons of estimated marginal means revealed no significant differences in MPR between males and females within NM, IM, and UM. For PM, sex-specific contrasts could not be estimated due to sparse data.

In parallel, including the CYP2D6 AS, the inclusion of a mixed-effects modeling approach was supported (random intercept variance = 9.20, SD = 3.03; residual variance = 0.09, SD = 0.30). Type III ANOVA revealed a significant main effect of CYP2D6 AS (F(7, 61.7) = 6.70, *p* < 0.001), whereas no significant main effects of sex, age, or CPR were observed. The interaction between sex and CYP2D6 AS was not significant. Examination of the fixed-effect estimates indicated that, compared with AS2, AS0, AS0.25, AS0.5, AS1, and AS1.5 exhibited a significantly lower MPR (AS0: β = −4.3, *p* = 0.03, AS0.25: β = −4.8, *p* = 0.01; AS0.5: β = −6.4, *p* = 0.006; AS1: β = −5.4, *p* < 0.001; AS1.5: β = −4.2, *p* = 0.003), whereas AS3 exhibited a higher MPR (β = 8.1, *p* = 0.01). Sex-specific pairwise comparisons of estimated marginal means revealed no significant differences in MPR between males and females within different AS groups (AS2, AS0.5, AS1). For AS ≥ 3, 0, 0.25, and 3, sex-specific contrasts could not be estimated due to sparse data.

For details on *p*- and β-values, see Table 4. Differences between subgroup numbers reported in Table 3 and Table 4 reflect differences between descriptive sample counts and model-based analyses after exclusion of outliers and handling of repeated observations.

### 3.3. Risperidone

For patients treated with risperidone, a detailed overview of the MPR in male and female patients, stratified by phenotype, is presented in Table 3. Detailed results for the CYP2D6 activity score subgroup are provided in Supplementary Table S2.

In the analysis including the CYP2D6 phenotype, as each sample was represented by a single observation in the final dataset, the random-effects structure was not identifiable. Therefore, a fixed-effects linear model was used for the analysis. ANOVA revealed no significant main effect of CYP2D6 phenotype, sex, the interaction, age, or CPR. Consistent with the ANOVA results, none of the confounders reached statistical significance. Sex-specific pairwise comparisons of estimated marginal means revealed no significant differences in MPR between males and females within NM or IM. For PM, sex-specific contrasts could not be estimated due to sparse data.

In parallel, including the CYP2D6 AS, ANOVA revealed no significant main effect of CYP2D6 AS, sex, the interaction, age, or CPR. Consistent with the ANOVA results, none of the confounders reached statistical significance. Sex-specific pairwise comparisons of estimated marginal means revealed no significant differences in MPR between males and females within different AS groups. For AS = 0, sex-specific contrasts could not be estimated due to sparse data.

For details on *p*- and β-values, see Table 4.

### 3.4. Escitalopram

For patients treated with escitalopram, a detailed overview of CD in male and female patients, stratified by phenotype, is presented in Table 3.

**Figure 1:**
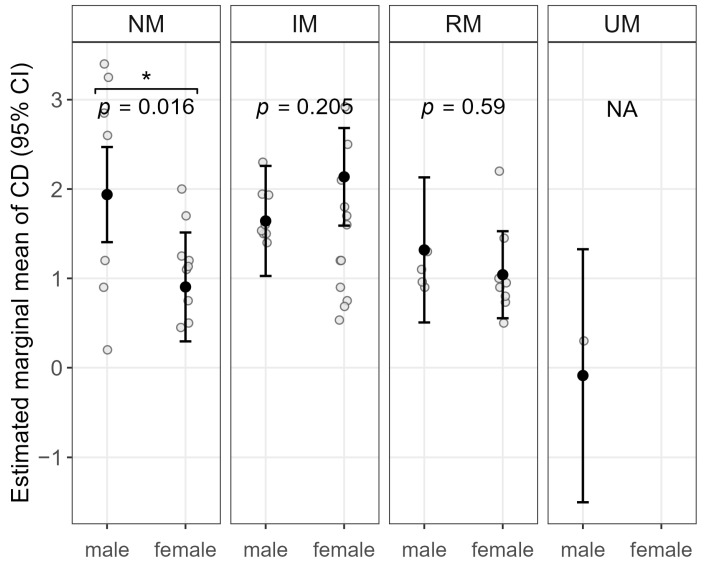
Sex-specific and CYP2C19 genotype-dependent differences in CD of escitalopram. Individual data points together with estimated marginal means (±95% confidence intervals) of CD stratified by sex and CYP2C19 phenotype, derived from a linear mixed-effects model including age and CPR as covariates and a random intercept for the patient. A significant sex difference was observed among CYP2C19 NM, with males exhibiting higher CD than females (*p* = 0.016). No significant sex-related differences were detected among IM or RM. Sex-specific estimates for UM could not be obtained due to sparse data. NM, normal metabolizer; IM, intermediate metabolizer; RM, rapid metabolizer; UM, ultrarapid metabolizer; na, not available; *p*, *p*-value; *, significant result.

In the analysis including the CYP2C19 phenotype, the random intercept variance indicated substantial between-subject variability (variance = 0.42, SD = 0.65), which clearly exceeded the residual variance (variance = 0.02, SD = 0.14), supporting the inclusion of a mixed-effects modeling approach. Type III ANOVA revealed a significant main effect of the CYP2C19 phenotype (F(3, 27.82) = 4.16, *p* = 0.015), the interaction between CYP2C19 phenotype and sex (F(2, 27.76) = 3.79, *p* = 0.035), as well as of age (F(1, 30.55) = 4.35, *p* = 0.045). No significant effect of CPR was observed. Examination of fixed-effect estimates indicated that the association between sex and CD differed by CYP2C19 phenotype. Sex-specific pairwise comparisons of estimated marginal means revealed a significant sex difference in CD among CYP2C19 NM, with males exhibiting higher CD than females (β = 1.01, *p* = 0.016). No significant sex differences were observed among IM or RM. Sex-specific contrasts could not be estimated for UM due to sparse data (Figure 1).

For details on *p*- and β-values, see Table 4.

Given prior evidence that smoking may affect escitalopram pharmacokinetics [23], we conducted an exploratory sensitivity analysis with available smoking data (N = 38 (13 male, 25 female; 26 nonsmokers, 12 smokers; 11 NM, 18 IM, 9 RM)), adjusted for the same covariates as in the main model. Smoking status was not significantly associated with dose-adjusted escitalopram concentrations. The sex-by-CYP2C19 interaction observed in the primary model was attenuated in this reduced dataset after inclusion of smoking, which may reflect reduced statistical power in the sensitivity analysis (Supplementary Material S3).

## 4. Discussion

The present study examined potential genotype-dependent sex differences in CYP2D6 and CYP2C19 metabolic capacity within genetically stratified metabolizer groups during routine treatment with venlafaxine, risperidone, and escitalopram. Preliminary evidence for higher metabolic capacity in CYP2C19 NM females treated with escitalopram was observed in this exploratory analysis.

Although genotype-based studies have demonstrated the influence of CYP genotype on pharmacokinetics, efficacy, and adverse effects [13,14,15,24,25,26], potential genotype-dependent sex differences in CYP2D6 and CYP2C19 metabolic capacity have rarely been systematically explored in previous clinical pharmacokinetic studies.

Among psychopharmacological drugs, previous studies have shown that CYP2D6 genotype/phenotype is an important determinant of venlafaxine metabolism [14,15,24]. These studies primarily focused on the functional impact of CYP2D6 genotype/phenotype and did not examine genotype-dependent sex differences. In addition, there are studies describing higher dose-corrected venlafaxine serum concentrations in females; however, without considering geno- or phenotypes [9,27].

Our findings, taking both factors (sex and CYP2D6) into consideration, support the previous literature on the predominant role of CYP2D6 for venlafaxine pharmacokinetics (lower MPR with reduced-activity phenotype, higher MPR with increased-activity phenotype), but do not suggest an additional significant effect of sex within genotype-defined subgroups.

Nevertheless, in our sample, the metabolic capacity for venlafaxine in females within NM and the corresponding AS groups (1.5, 2) was 79.52%, 65.66%, and 71.80% that of men; however, these differences were not statistically significant, possibly due to limited sample size (N = 57, N = 11, N = 46). In line, Kashuba et al. also reported that the dextromethorphan metabolic ratio (parent/metabolite) in CYP2D6 NM men was approximately 73% of that in women, indicating also a lower metabolic capacity in females; however, this result also was not statistically significant due to limited sample size (N = 19; to detect a 30% difference, 75 male and 75 female patients would have been needed) [28].

Similarly, the influence of CYP2D6 genetic variation on risperidone pharmacokinetics, as well as effects on drug tolerability, is well established [29,30,31,32,33]. The impact of CYP2D6 on MPR is mostly relevant for PM but also shown for IM [29]. In our cohort, this association could not be confirmed, most likely due to the limited sample size (only 1 PM included). To date, no data are available focusing on genotype-dependent sex differences in the metabolic capacity for risperidone. In line with the findings for venlafaxine, no sex-specific differences for risperidone were observed in our sample.

Previous studies on sex differences in CYP2D6 activity have been inconsistent. For example, one study reported lower CYP2D6 activity in PM females but not in PM men compared to a NM reference group (probe drug berberine) [34], another study reported a slightly higher CYP2D6 activity in women of childbearing age compared to men (probe drug dextromethorphan); however, they emphasized that the differences were moderate and influenced by hormonal status and contraceptive use [35]. Furthermore, a third study did not observe differences in mean CYP2D6 activity between men, women taking oral contraceptives, and regularly menstruating women using the same probe drug [36]. Taken together, these findings suggest that sex-specific differences in CYP2D6 activity, if present, are generally small and context-dependent.

In our sample, the CD for escitalopram among CYP2C19 NM was higher in males compared to females, indicating lower metabolic capacity in males. For escitalopram, the impact of CYP2C19 on serum concentrations, as well as on drug tolerability, is well known [13,37,38]. However, sex-specific information was lacking so far. Our findings suggest that sex-associated differences in metabolic capacity may become apparent within the NM subgroup treated with escitalopram. If confirmed in larger and independent cohorts, higher escitalopram exposure in male CYP2C19 normal metabolizers could contribute to interindividual variability in systemic drug exposure. Increased exposure may be associated with a higher risk of dose-dependent adverse effects, whereas lower exposure could potentially be associated with reduced antidepressant efficacy in the absence of dose adjustment. However, given the exploratory nature of the present findings and the small subgroup size, no direct dosing recommendations can be derived, and the clinical relevance remains to be established in adequately powered studies. Therefore, this finding should nevertheless be interpreted cautiously as preliminary results and regarded as hypothesis-generating.

Supporting our results, previous studies have also suggested that sex-related differences in CYP2C19 activity may occur. In a Korean sample, however, contrasting our results, CYP2C19 genotype-derived NM men showed lower metabolic ratios of omeprazole/5-hydroxyomeprazole than women, indicating higher enzyme activity in men, whereas no sex differences were reported for a Swedish sample [39]. Data also suggest that CYP2C19 activity may be affected by oral contraceptives, with inhibition of CYP2C19 reported in some studies [40,41], but potentially only in *1/*1 carriers [26]. These findings, including ours, indicate that sex-related differences in CYP2C19 activity are likely modest and may depend on hormonal influences and population-specific factors.

To summarize, we found preliminary evidence for sex-specific metabolic capacity of CYP2C19 in NM patients treated with escitalopram. This finding suggests that sex-dependent effects on metabolic capacity may be most relevant in individuals with normal enzyme activity, while extreme genotype-defined metabolizer phenotypes (e.g., UM and PM) may override more subtle sex-associated differences. However, while the present results offer initial indications, the limited sample size necessitates a cautious interpretation and underscores the need for replication in larger samples.

### Strengths and Limitations

A key strength of this study lies in the use of routinely collected data from TDM, resulting in findings of high clinical relevance and external validity. The combination of TDM data with pharmacogenetic information on CYP2D6 and CYP2C19 phenotypes enables a differentiated analysis of genetic and non-genetic factors influencing the pharmacokinetics of psychotropic drugs under real-world conditions. In addition, model-based statistical analyses tailored to the underlying data structure were applied. Linear mixed-effects models were employed to account for inter-individual variability and repeated measurements where present. Fixed effects and interactions were evaluated using Type III analysis of variance, complemented by adjusted post hoc comparisons based on estimated marginal means. This integrated approach enabled robust inference while providing detailed, interpretable estimates of genotype- and sex-specific effects. A further methodological advantage is the systematic consideration of physiological confounders through the inclusion of the creatinine production rate (CPR) as a surrogate parameter for fat-free mass. This addresses a frequently neglected influencing factor in sex-specific pharmacokinetic analyses and strengthens the physiological interpretation of the results. Furthermore, the exclusion of relevant moderate and strong enzyme inhibitors or inducers reduced the risk of confounding due to phenoconversion. Finally, the inclusion of two independent university cohorts increases the generalizability of the results within the psychiatric care context. As patients were hospitalized, adherence can be assumed to be high, steady-state conditions were likely reached, and the correct sampling material (serum tubes without gel) and time (through levels) were used for concentration measurements. The same laboratory was used for the analysis of both hospitals to decrease inter-laboratory variability.

Despite the aforementioned strengths, the study has several limitations. Owing to its retrospective, observational design, causal relationships cannot be inferred, and residual confounding may persist despite statistical adjustment. The number of cases in individual, highly stratified subgroups was limited, particularly for PM and UM categories (e.g., venlafaxine: PM N = 4, UM N = 4; risperidone: PM N = 1; escitalopram: UM N = 1) as well as for several CYP2D6 activity score subgroups (e.g., venlafaxine: AS0.25 N = 3, AS0.5 N = 6, AS ≥ 3 N = 2; risperidone: AS0.25 N = 2, AS0.5 N = 2). In addition, some CYP2C19 phenotype groups in the escitalopram sample were also represented by only small numbers of observations (e.g., NM N = 16, RM N = 12, UM N = 1). As a result, some phenotype categories were based on only very few observations, and corresponding estimates should be interpreted with caution. While the overall analyses confirmed the expected effects of genotype on pharmacokinetic parameters, the limited subgroup sizes reduced the ability to reliably detect more subtle sex-specific differences within genotype-defined metabolizer groups. This particularly applies to the interaction analyses and may have increased the risk of both Type II error and unstable subgroup-specific estimates. The subgroup analyses should therefore be regarded as exploratory and hypothesis-generating, requiring confirmation in larger and adequately powered samples. Additionally, the analysis is based on pharmacokinetic surrogate parameters (MPR and CD), which, while established in clinical practice, can be influenced by factors such as treatment adherence, the time of blood sampling, dosage adjustments, or differences in volume of distribution. Furthermore, different pharmacokinetic surrogate parameters were used across the investigated drugs. For venlafaxine and risperidone, MPRs were applied as established markers of CYP2D6-mediated metabolic conversion. For escitalopram, CD was used as no corresponding metabolite data were consistently available. These metrics capture related but not identical pharmacokinetic processes. MPRs primarily reflect enzymatic conversion capacity, whereas CD is influenced by absorption, distribution, metabolism, and elimination processes. Similarly, CPR represents an indirect surrogate marker of fat-free mass and is influenced by sex, body composition, renal function, and creatinine generation. As a consequence, adjustment for CPR may partially account for variance that is also related to sex and may introduce some degree of collinearity between covariates. However, CPR was included to reduce confounding by body composition, which is a relevant determinant of pharmacokinetic variability. This represents a trade-off between confounding control and potential overadjustment in the present analyses. Several factors known to influence CYP enzyme activity could not be comprehensively assessed, including menopausal status, menstrual cycle phase, inflammatory status, and hepatic function. Information on smoking status was available only for a subset of patients and was therefore not included in the primary analyses due to missing data and the resulting loss of statistical power. Information on concomitant medication was available (for a full list, see Supplementary Material, Supplemental Table S4); however, only four patients received oral contraceptives or estradiol treatment, precluding meaningful evaluation of their effects. Although major CYP inhibitors and inducers were considered, the overall burden of concomitant medication could not be fully accounted for. Consequently, residual confounding by unmeasured physiological and environmental factors cannot be excluded. Finally, differences in the indication spectrum and treatment practices between the two study centers may have introduced additional heterogeneity into the analysis. However, these factors are unlikely to have substantially affected the pharmacokinetic analyses.

## 5. Conclusions

To advance the understanding of the relative contributions of sex and genetics to the pharmacokinetics of psychotropic drugs, we investigated genotype-dependent sex differences in CYP2D6 and CYP2C19-related metabolic capacity during routine psychotropic drug treatment. We observed preliminary evidence of higher apparent CYP2C19-related metabolic capacity in a small subgroup of female CYP2C19 NM patients treated with escitalopram compared with males. These findings suggest that sex-dependent effects on apparent CYP2C19-related metabolic capacity may be more apparent in individuals with normal enzyme activity, whereas pronounced genotype-defined metabolizer phenotypes such as UM and PM may mask more subtle sex-associated differences. Given the limited sample size, this finding should be interpreted with caution, considered hypothesis-generating, and requires confirmation in larger, adequately powered studies.

## Figures and Tables

**Table 1:** Demographic data of the patients included in the sample. Baseline characteristics were defined using the first available sample per patient, as some individuals contributed more than one sample.

	Combined Sample		Wuerzburg Sample		Frankfurt Sample	
	N	Mean ± SD (Range)	N	Mean ± SD (Range)	N	Mean ± SD (Range)
Included patients	179		134		45	
Age (years)	179	46.3 ± 15.03 (18–80)	134	47.27 ± 15.08 (18–80)	45	43.4 ± 14.67 (19–69)
Male/Female	86/93		63/71		23/22	
Nonsmoker/Smoker	76/58		55/36		21/22	
Creatinine (mg/dL)	179	0.91 ± 0.2 (0.47–1.4)	134	0.95 ± 0.19 (0.54–1.4)	45	0.82 ± 0.19 (0.47–1.36)
GFR	179	87.6 ± 17.6 (49–>120)	134	85.4 ± 18 (49–122)	45	95.1 ± 13.8 (62–>120)
Number of concomitant medications (yes/no)	173/6	5.23 ± 3.5 (0–16)	129/5	4.69 ± 3.37 (1–16)	44/1	6.84 ± 3.42 (0–15)
**CYP2D6**						
NM/IM/PM/UM (%)	95/56/8/4 (53.1/31.3/4.5/2.2)		69/47/5/1 (51.1/35.1/3.7/0.7)		26/9/3/3 (57.8/20.0/6.7/6.7)	
AS 0/0.25/0.5/1/1.25/1.5/2/3/≥3 (%)	8/5/7/36/2/17/76/2/1 (4.5/2.8/3.9/20.1/1.1/9.0/ 42.5/1.1/0.6)		5/5/4/30/2/9/58/1 (3.7/3.7/3.0/22.4/1.5/ 6.7/43.3/0.7)		3/0/3/6/0/8/18/2/0 (6.7/0/6.7/13.3/0/17.8/ 40/4.4)	
CYP2D6 affecting comedication No/Yes Inhibitors (w/m/s)	127/52 (46/4/8)		93/41 (35/3/8)		34/11 (11/1/0)	
**CYP2C19**						
NM/IM/PM/RM/UM (%)	80/39/1/52/7 (44.7/21.8/0.6/29.1/3.9)		62/27/1/39/5 (46.3/20.1/0.7/29.1/3.7)		18/12/0/13/2 (40.0/26.7/0/28.9/4.4)	
CYP2C19 affecting comedication No/Yes Inhibitors (w/m) Inducers (N)	141/38 (34/2) (2)		98/36 (34/2) (0)		43/2 (0) (2)	

N, number of patients; (%), percentage number; SD, standard deviation; GFR (CKD-EPI, mL/min/1.73 m^2^); NM, normal metabolizer; IM, intermediate metabolizer; PM, poor metabolizer; RM, rapid metabolizer; UM, ultrarapid metabolizer; AS, Activity Score; w, weak; m, moderate; s, strong.

**Table 2:** Demographic characteristics of the analyzed drug-specific samples before and after exclusion of relevant comedication, with repeated TDM observations possible for individual patients.

	Venlafaxine		Risperidone		Escitalopram	
	N	Mean ± SD	N	Mean ± SD	N	Mean ± SD
Included samples	117		73		51	
Age [years]	117	46.76 ± 14.89	73	48.68 ± 14.23	51	42.37 ± 13.78
Male/Female	57/60		34/39		20/31	
Nonsmoker/Smoker	45/49		33/19		26/13	
Comedication No/Yes Inhibitors (weak/moderate/strong)	107/10 (3/1/6)		59/14 (8/4/2)		43/8 (7/1/0)	
	N		N		N	
Included samples after exclusion of relevant concomitant medications	110		67		50	
CYP2D6 NM/IM/PM/UM	57/34/4/4		36/25/1/0			
CYP2D6 Activity Score 0/0.25/0.5/1/1.25/1.5/2/3	4/3/7/18/0/11/46/1		1/2/2/15/2/4/30/0			
CYP2C19 NM/IM/PM/RM/UM					16/21/0/12/1	

N, number of samples; SD, standard deviation; NM, normal metabolizer; IM, intermediate metabolizer; PM, poor metabolizer; RM, rapid metabolizer; UM, ultrarapid metabolizer.

**Table 3:** MPR (venlafaxine and risperidone) and CD (escitalopram) values (mean ± standard deviation) of TDM samples after exclusion of relevant comedication and outliers, stratified by sex and genetically defined metabolizer phenotypes. Repeated observations from individual patients were possible.

	Venlafaxine (CYP2D6)				Risperidone (CYP2D6)				Escitalopram (CYP2C19)			
	N (m/f)	MPR Male (Mean ± SD)	MPR Female (Mean ± SD)	Female Metabolic Capacity [%] Compared to Male	N (m/f)	MPR Male (Mean ± SD)	MPR Female (Mean ± SD)	Female Metabolic Capacity [%] Compared to Male	N (m/f)	CD Male (Mean ± SD)	CD Female (Mean ± SD)	Female Dose-Corrected Exposure [%] Compared to Male
NM	57 (30/27)	6.25 ± 4.34	4.97 ± 3.87	79.52	25 (7/18)	2.9 ± 1.87	3.99 ± 3.68	137.59	16 (7/9)	2.06 ± 1.27	1.77 ± 2.11	85.92
IM	34 (14/20)	1.97 ± 1.3	2.16 ± 1.79	109.64	25 (11/14)	3.36 ± 2.91	2.49 ± 2.48	74.11	21 (8/13)	1.71 ± 0.31	1.72 ± 1.10	100.58
PM	4 (0/4)		1.14 ± 0.77		1 (0/1)		0.57					
RM									12 (4/8)	1.06 ± 0.18	1.07 ± 0.53	100.94
UM	4 (2/2)	9.14 ± 2.63	9.37 ± 6.14	102.53					1 (1/0)	0.30		

N, number of samples; m, male; f, female; MPR, metabolite-to-parent ratio; CD, dose-corrected concentration; NM, normal metabolizer; IM, intermediate metabolizer; PM, poor metabolizer; RM, rapid metabolizer; UM, ultrarapid metabolizer.

**Table 4:** Linear mixed-effects models investigating the association between MPR (venlafaxine and risperidone), or CD (escitalopram) as surrogates for metabolic capacity (enzyme activity) and sex in analyzed TDM samples, adjusted for age and creatinine production rate (CPR). Analyses were based on the final model-specific datasets. Sex-specific contrasts within CYP2D6 and CYP2C19 phenotype groups were obtained using estimated marginal means (emmeans) for mixed-effects models. β(MPR) and β(CD) represent male-to-female contrasts derived from the model-based comparisons. Positive β values indicate higher MPR or CD in males compared with females. *p*-values represent Holm-adjusted model-based sex contrasts within metabolizer groups. Corresponding 95% confidence intervals (CI) are reported. Significant results are shown in bold.

	Venlafaxine (CYP2D6)					Risperidone (CYP2D6)					Escitalopram (CYP2C19)				
	N	β (MPR)	SE	*p*-Value	95% CI	N	β (MPR)	SE	*p*-Value	95% CI	N	β (CD)	SE	*p*-Value	95% CI
NM 1.25 1.5 2	52 0 11 41	0.86 1.03 1.81	1.37 2.62 1.27	0.53 0.69 0.16	−1.88–3.59 −4.19–6.25 0.75–4.37	25 2 4 19	1.14 −1.52 1.52 −1.90	2.02 6.13 5.58 4.67	0.57 0.81 0.79 0.69	−2.95–5.24 −14.21–11.16 −10.02–13.05 −11.56–7.76	16	1.01	0.39	**0.02**	0.21–1.81
PM 0	4 4					1 1					0				
IM 0.25 0.5 1	26 3 6 17	−0.42 0.56 −0.90	1.59 3.17 1.71	0.79 0.86 0.60	−3.58–2.74 5.76–6.89 −4.31–2.50	17 2 2 13	1.49 −1.14 1.85 −1.78	1.92 5.94 5.25 4.40	0.44 0.85 0.73 0.69	−2.41–5.39 −13.44–11.15 −9.02–12.71 −10.89–7.33	21	−0.53	0.40	0.20	−1.35–0.30
RM											12	0.25	0.46	0.59	−0.69–1.19
UM 3	2 1	−0.72	4.11	0.86	−8.90–7.46						1				

N, number of samples; β (MPR), regression coefficient of MPR (male vs. female); β (CD), regression coefficient of CD (male vs. female); MPR, metabolite-to-parent ratio; CD, concentration-to-dose ratio; SE, standard error; CI, confidence interval; NM, normal metabolizer; PM, poor metabolizer; IM, intermediate metabolizer; RM, rapid metabolizer; UM, ultrarapid metabolizer.

## Data Availability

Data are available on request from the corresponding author.

## Supplementary Materials

The following supporting information can be downloaded at: https://www.mdpi.com/article/10.3390/genes17060718/s1. S1: Therapeutic Drug Monitoring and Genotyping; Table S1: Haplotype table giving the combination of the SNPs for the according haplotypes. S2: Table S2: MPR and standard deviations of TDM-samples after exclusion of relevant comedication and outliers, stratified by sex and genetically defined metabolizer phenotypes; S3: Sensitivity Analysis Including Smoking Status in the Escitalopram Sample; Supplemental Table S3: Sensitivity analysis of escitalopram CD including smoking status as an additional covariate. Analysis restricted to samples with available smoking information (N = 38). Linear mixed-effects model adjusted for age, CPR, smoking status, CYP2C19 phenotype, and the interaction between sex and CYP2C19 phenotype. β(CD) values represent male-to-female contrasts estimated using emmeans. Positive β values indicate higher CD values in males. S4: Table S4: Alphabetical list of concomitant medications reported in the study cohort. References [6,23,42,43,44,45] are cited in the supplementary materials.

## Abbreviations

The following abbreviations are used in this manuscript:

ANOVA	analysis of variance
AS	activity score
CD	dose-corrected serum concentration
CPIC	Clinical Pharmacogenetics Implementation Consortium
CPR	creatinine production rate
CYP	cytochrome P450 enzymes
FFM	fat-free mass
IM	intermediate metabolizers
MPR	metabolite-to-parent ratio
NM	normal metabolizers
PM	poor metabolizers
RM	rapid metabolizers
TDM	therapeutic drug monitoring
UM	ultrarapid metabolizers
