# Supplementary material for: Preliminary Evidence for Sex Differences in CYP2C19 Metabolic Capacity During Psychotropic Drug Treatment
Source: Genes (Basel). 2026 Jun 21;17(6):718. doi: 10.3390/genes17060718 (PMC13299576; doi:10.3390/genes17060718)
Supplement: Supplementary file 1 [file genes-17-00718-s001.zip › genes-4347449-supplementary.pdf]

Supplementary Material

To

Preliminary Evidence for Sex Differences in CYP2C19 Metabolic Capacity During Psychotropic Drug Treatment

Janina Eiberger[1], Heike Weber[1], Andreas Reif[2], Jürgen Deckert[1,3], Sebastian Walther[1], Martina Hahn[2,4]#*, Maike Scherf-Clavel[1]#

[1] Department of Psychiatry, Psychosomatics and Psychotherapy, Center of Mental Health, University Hospital of Würzburg, 97080 Würzburg, Germany

[2] Department of Psychiatry, Psychosomatics and Psychotherapy, University Hospital Frankfurt, 60528 Frankfurt, Germany

[3] Institute of Clinical Epidemiology and Biometry, Julius-Maximilians-Universität Würzburg, Würzburg, Germany

[4] Department of mental health, varisano Hospital Frankfurt Hoechst, 65929 Frankfurt, Germany

#Shared last authorship

* Correspondence: Prof. Martina Hahn; Universitätsklinikum Frankfurt, Klinik für Psychiatrie, Psychosomatik und Psychotherapie, Heinrich-Hoffmann-Strasse 10; 60590 Frankfurt am Main; Germany; M.hahn@med.uni-frankfurt.de; Tel.: 049-69-6301-4867

Table of Contents

S1: Therapeutic Drug Monitoring and Genotyping

Supplemental Table S2 MPR and standard deviations of TDM-samples after exclusion of relevant comedication and outliers, stratified by sex and genetically defined metabolizer phenotypes

S3: Sensitivity Analysis Including Smoking Status in the Escitalopram Sample

Supplemental Table S4 Alphabetical list of concomitant medications reported in the study cohort.

S1: Therapeutic Drug Monitoring and Genotyping

Genotyping of CYP2D6 and CYP2C19 and TDM for both cohorts was performed at the Department of Psychiatry, Psychosomatics and Psychotherapy of the University Hospital of Würzburg.

Therapeutic drug monitoring was performed according to the Arbeitsgemeinschaft für Neuropsychopharmakologie und Pharmakopsychiatrie (AGNP)-TDM expert group consensus guideline [1]. Blood was drawn at trough concentrations at steady-state [1]. Serum concentrations of the drugs and their metabolites were determined using liquid chromatography tandem mass spectrometry (LC-MS/MS) methods using MassTox® TDM Serie A, provided by Chromsystems (Chromsystems Instruments & Chemicals GmbH, Gräfeling, Germany) [2]. The laboratory was certified by a quality control program [2].

Genotyping of CYP2D6 and CYP2C19 variants, including single nucleotide polymorphisms (SNPs) and star alleles (Supplemental Table S4), was carried out using a MassARRAY Analyzer 4 platform (Agena Bioscience GmbH, Hamburg, Germany). Analyses were performed with a custom-designed assay panel employing SpectroCHIP®-96 Arrays in combination with iPLEX® Pro chemistry according to the manufacturer's protocol. Because of limited assay specificity, rs3892097, rs16947, and rs1080995 were additionally analysed by restriction fragment length polymorphism (RFLP) analysis. Primer sequences can be provided upon request. Copy number variations (CNVs) were assessed using the CYP2D6 RealFast™ CNV Assay (ViennaLab Diagnostics GmbH, Vienna, Austria) [4]. Hybrid alleles and complex CYP2D6 structural rearrangements were not systematically assessed by the applied genotyping approach. The laboratory participated successfully in an external quality assurance program [3]. Haplotype assignment for all analysed SNPs was based on the gene-specific allele definitions provided by the PharmVar database (https://www.pharmvar.org/genes; Supplemental Table S4). CYP2D6 and CYP2C19 phenotypes were classified in accordance with the recommendations of the Clinical Pharmacogenetics Implementation Consortium (CPIC) [5].

Supplemental Table S1 Haplotype table giving the combination of the SNPs for the according haplotypes.

	HAPLOTYPE											
CYP2C19		**rs12248560**	**rs28399504**	**rs12769205**	**rs58973490**	**rs4986893**						
	*1	C	A	A	G	G						
	*2A	C	A	G	G	G						
	*3	C	A	A	G	A						
	*4A	C	G	A	G	G						
	*4B	T	G	A	G	G						
	*11	C	A	A	A	G						
	*17	T	A	A	G	G						
CYP2D6		**rs1080985**	**rs28735595**	**rs1065852**	**rs1080995**	**rs28371706**	**rs5030655**	**rs3892097**	**rs35742686**	**rs5030656**	**rs16947**	**rs1135840**
	*1	G	T	G	C	G	A	G	T	I	G	C
	*2A	C	C	G	G	G	A	G	T	I	A	G
	*2B	G	T	G	C	G	A	G	T	I	A	G
	*2C	C	C	G	G	G	A	G	T	I	G	G
	*3	G	T	G	C	G	A	G	D	I	G	C
	*4	G	T	A	C	G	A	A	T	I	G	G
	*4J	G	T	A	C	G	A	A	T	I	G	C
	*4K	G	T	A	C	G	A	A	T	I	A	G
	*4M	G	C	G	C	G	A	A	T	I	G	C
	*4N	G	C	A	C	G	A	A	T	I	G	G
	*4P	G	C	A	C	G	A	A	T	I	G	C
	*5					Complete Gene Deletion						
	*6A	G	T	G	C	G	D	G	T	I	G	C
	*6C	G	T	G	C	G	D	G	T	I	G	G
	*9	G	T	G	C	G	A	G	T	D	G	C
	*10A	G	T	A	C	G	A	G	T	I	G	G
	*10B	G	C	A	C	G	A	G	T	I	G	G
	*10C	G	C	A	C	G	A	G	T	I	G	C
	*14	G	T	G	G	G	A	G	T	I	A	G
	*17	G	T	G	C	A	A	G	T	I	A	G

*34	G	T	G	C	G	A	G	T	I	A	C
*35A	C	T	G	C	G	A	G	T	I	A	G
*35B	C	C	G	C	G	A	G	T	I	A	G
*39	G	T	G	C	G	A	G	T	I	G	G
*41	G	C	G	G	G	A	G	T	I	A	G
*46A	G	C	G	C	G	A	G	T	I	A	G
*46B	G	C	G	C	G	A	G	T	I	G	G
*46C	G	C	G	C	G	A	G	T	I	G	C
*58	G	C	G	G	A	A	G	T	I	A	G
*64	G	C	A	C	A	A	G	T	I	G	G
*69	G	C	A	C	G	A	G	T	I	A	G
*71	C	T	G	C	G	A	G	T	I	G	C
*82	G	T	G	C	T	A	G	T	I	G	C
*88	G	T	G	G	G	A	G	T	I	G	G
*114	G	T	A	C	G	A	G	T	I	A	G

References

[1] Hiemke C, Bergemann N, Clement HW et al. Consensus guidelines for therapeutic drug monitoring in neuropsychopharmacology: update 2017. Pharmacopsychiatry 2018; 51: 9-62

[2] Chromsystems Instrumental & Chemicals GmbH. Effizientes Drug Monitoring mit LC-MS/MS. www.Chromsystems.de. 2019

[3] INSTAND Gesellschaft zur Förderung der Qualitätssicherung in medizinischen Laboratorien e. V. 2020. https://www.instand-ev.de/ueber-instand-ev/instand-ev.html; Accessed 10 Feb, 2020

[4] ViennaLab Diagnostics GmbH. CYP2D6 RealFast CNV Assay. 2021

[5] CPIC - Clinical Pharmacogenetics Implementation Consortium. 2021. https://cpicpgx.org/; Accessed 28 May, 2026

Supplemental Table S2

Supplemental Table S2 MPR and standard deviations of TDM-samples after exclusion of relevant comedication and outliers, stratified by sex and genetically defined metabolizer phenotypes. Repeated observations from individual patients were possible.

	Venlafaxine (CYP2D6)				Risperidone (CYP2D6)			
	N (m/f)	MPR male (mean±SD)	MPR female (mean±SD)	Female metabolic capacity [%] compared to male	N (m/f)	MPR male (mean±SD)	MPR female (mean±SD)	Female metabolic capacity [%] compared to male
AS1.25					2 (1/1)	1.29	1.12	86.82
AS1.5	12 (9/3)	3.29 ± 2.23	2.16 ± 0.52	65.55	5 (1/4)	5.62	2.21 ± 1.54	39.32
AS2	45 (21/24)	7.41 ± 4.38	5.32 ± 3.96	71.80	18 (5/13)	2.68 ± 1.64	4.83 ± 4.05	180.22
AS0.25	3 (0/3)	0.95 ± 0.61	0.87 ± 0.64	91.58	3 (2/1)	5.68 ± 6.13	0.19	3.35
AS0.5	6 (4/2)	2.20 ± 1.01	0.45 ± 0.57	20.45	3 (2/1)	2.31 ± 2.23	0.90	28.96
AS1	19 (7/12)	1.92 ± 1.18	2.69 ± 2.01	140.10	15 (5/10)	2.54 ± 1.99	3.16 ± 2.66	124.41
AS0	4 (0/4)		1.14 ± 0.77		1(0/1)		0.57	
AS3	4 (2/2)	9.14 ± 2.63	9.37 ± 6.14	102.53				

N, number of samples; m, male; f, female; MPR, metabolite-to-parent ratio; AS, Activity Score

S3: Sensitivity Analysis Including Smoking Status in the Escitalopram Sample

Methods

As smoking has previously been reported to influence escitalopram pharmacokinetics (Scherf-Clavel, Deckert et al. 2019), a sensitivity analysis was conducted in the subgroup of patients with available smoking status information. The same linear mixed-effects model as in the primary escitalopram analysis was applied, including sex, CYP2C19 phenotype, age, CPR, and smoking status as fixed effects, as well as the interaction between sex and CYP2C19 phenotype. Smoking status was included as an additional covariate. Patients with missing smoking information were excluded.

Results

After exclusion of samples with missing smoking status, 38 escitalopram samples (13 male, 25 female; 26 nonsmokers, 12 smokers) were included in the sensitivity analysis. CYP2C19 phenotype distribution was NM (n=11), IM (n=18), and RM (n=9).
In the adjusted model including smoking status, CYP2C19 phenotype remained significantly associated with dose-corrected escitalopram concentrations (F=4.63, p=0.02). Smoking status was not significantly associated with concentrations (F=0.34, p=0.57), and age remained a significant covariate (F=4.91, p=0.04).
Importantly, adjustment for smoking status attenuated the sex-by-CYP2C19 interaction, which was no longer statistically significant, whereas it had reached significance in the primary analysis without smoking adjustment.

Supplemental Table S3 Sensitivity analysis of escitalopram CD including smoking status as an additional covariate. Analysis restricted to samples with available smoking information (N=38). Linear mixed-effects model adjusted for age, CPR, smoking status, CYP2C19 phenotype, and the interaction between sex and CYP2C19 phenotype. β(CD) values represent male-to-female contrasts estimated using emmeans. Positive β values indicate higher CD values in males.

	N (Nonsmoker/ Smoker)	β (CD)	SE	p-value	95% CI
NM	11 (9/2)	0.27	0.47	0.57	-0.72-1.27
IM	18 (13/5)	-0.68	0.43	0.13	-1.57-0.21
RM	9 (4/5)	0.65	0.56	0.26	-0.52-1.83

N, number of samples; (%), β(CD), regression coefficient of CD (male vs. female); CD, concentration-to-dose ratio; SE, standard error; CI, confidence interval; NM, normal metabolizer; IM, intermediate metabolizer; RM, rapid metabolizer

Discussion:

In a sensitivity analysis including smoking status as an additional covariate, the association between CYP2C19 phenotype and escitalopram concentrations remained stable, whereas smoking itself was not significantly associated with dose-corrected concentrations. Importantly, the sex-by-CYP2C19 interaction observed in the primary analysis was attenuated after adjustment for smoking.

This finding suggests that the observed interaction is sensitive to model specification and may be influenced by covariate structure and reduced sample size in the smoking-subgroup analysis. Given the limited number of patients with available smoking information, reduced statistical power is a

likely contributing factor. At the same time, residual confounding by smoking in the primary analysis cannot be excluded.

Overall, these results indicate that while the main effect of CYP2C19 phenotype appears robust, evidence for sex-specific differences in escitalopram exposure is less consistent and should be interpreted with caution. Further studies in larger cohorts with comprehensive characterization of smoking behavior are needed to clarify the stability of this interaction effect.

References:

Scherf-Clavel, M., et al. (2019). "Smoking Is Associated With Lower Dose-Corrected Serum Concentrations of Escitalopram." J Clin Psychopharmacol **39**(5): 485–488.

Supplemental Table S4

Supplemental Table S4 Alphabetical list of concomitant medications (generic drug names) reported in the study cohort.

Adalimumab	Macrogol
Agomelatine	Magnesium
Amiodarone	Mebeverine
Amitriptyline	Melatonin
Amlodipine	Metamizole
Amoxicillin	Metformin
Apixaban	Methantheline bromide
Asenapine	Methocarbamol
Aspirin (acetylsalicylic acid)	Methylphenidate
Atenolol	Methylprednisolone
Atorvastatin	Metoprolol
Betahistine	Mirtazapine
Bictegravir + Emtricitabine + Tenofovir alafenamide	Mometason
Biotin	Naltrexone
Bisoprolol	Nebivolol
Brinzolamide	Nicotine
Bromazepam	Omeprazole
Budesonide + Formoterol	Oxazepam
Butylscopolamine	Pantoprazole
Calcium	Paracetamol
Candesartan	Pentosan polysulfate sodium
Carvedilol	Perazine
Certolizumab	Pentosan polysulfate
Cetirizine	Pipamperone
Chlorprothixene	Piretanide
Clomipramine	Piribedil
Clonazepam	Potassium bicarbonate
Clonidine	Prednisolone
Dexamethasone + Neomycin + Polymyxin B	Pregabalin
Glycerol cream	Promethazine
Diazepam	Propiverine
Digestive enzymes	Propranolol
Digitoxin	Prothipendyl
Dimenhydrinate	Psyllium husk
Distigmine	Quetiapine
Domperidone	Rabeprazole
Duloxetine	Ramipril
Empagliflozin	Risperidone
Enoxaparin	Rivaroxaban
Escitalopram	Rotigotine
Esketamine	Sage extract
Esomeprazole	Salbutamol
Estradiol	Saxagliptin
Ethinylestradiol + Levonorgestrel	Selenium
Etilefrine	Sertraline
Etoricoxib	Simeticone
Ezetimibe	Simvastatin

Fentanyl	Sinupret (gentian root, vervain, sorrel, elderflower, primrose flowers)
Ferrous sulfate	Sitagliptin
Fingolimod	Sodium chloride
Fluticasone + Salmeterol	Sodium polystyrene sulfonate
Fluticasone + Umeclidinium + Vilanterol	Spironolactone
Folic acid	Sulpiride
Gabapentin	Sumatriptan
Ginkgo biloba extract	Tamsulosin
Haloperidol	Tapentadol
Hormonal contraceptive (unspecified)	Temazepam
Human insulin	Tiapride
Hydrochlorothiazide	Tilidine
Hydromorphone	Tiotropium
Ibuprofen	Tizanidine
Immunotherapy (unspecified)	Tolperisone
Indapamide	Tolterodine
Insulin glargine	Torasemide
Irbesartan	Trazodone
Iodide	Urapidil
Irbesartan	Valerian root extract (Valeriana officinalis)
Ivermectin	Valproate
Ketamine	Valsartan
Lactulose	Venlafaxine
Lamotrigine	Vitamin B6
Lavender oil	Vitamin B12
Leflunomide	Vitamin C
Lercandipine	Vitamin D
Levodopa + Carbidopa + Entacapone	Vitamin K
Levothyroxine	Zinc
Levothyroxine + Liothyronine	Zolpidem
Levothyroxine + Potassium iodide	Zopiclone
Lisdexamfetamine	
Lithium	
Loratadine	
Lorazepam	
